# Prevalence and persistence of *Neisseria meningitidis* carriage in Swedish university students

**DOI:** 10.1017/S0950268823000018

**Published:** 2023-02-13

**Authors:** Säll Olof, Eriksson Lorraine, Idosa Berhane A., Persson Alexander, Magnuson Anders, Thulin Hedberg Sara, Sundqvist Martin, Olcén Per, Fredlund Hans, Stenmark Bianca, Särndahl Eva, Mölling Paula, Jacobsson Susanne

**Affiliations:** 1 Department of Infectious Diseases, Faculty of Medicine and Health, Örebro University, Örebro, Sweden; 2 Department of Laboratory Medicine, Clinical Microbiology, Faculty of Medicine and Health, Örebro University, Örebro, Sweden; 3 Faculty of Medicine and Health, Inflammatory Response and Infection Susceptibility Centre (iRiSC), School of Medical Sciences, Örebro University, Örebro, Sweden; 4 Clinical Epidemiology and Biostatistics, School of Medical Sciences, Örebro University, Örebro, Sweden

**Keywords:** Carriage, *Neisseria meningitidis*, Swedish snus, university students, whole genome sequencing

## Abstract

The bacterium *Neisseria meningitidis* causes life-threatening disease worldwide, typically with a clinical presentation of sepsis or meningitis, but can be carried asymptomatically as part of the normal human oropharyngeal microbiota. The aim of this study was to examine *N. meningitidis* carriage with regard to prevalence, risk factors for carriage, distribution of meningococcal lineages and persistence of meningococcal carriage. Throat samples and data from a self-reported questionnaire were obtained from 2744 university students (median age: 23 years) at a university in Sweden on four occasions during a 12-month period. Meningococcal isolates were characterised using whole-genome sequencing. The carriage rate among the students was 9.1% (319/3488; 95% CI 8.2–10.1). Factors associated with higher carriage rate were age ≤22 years, previous tonsillectomy, cigarette smoking, drinking alcohol and attending parties, pubs and clubs. Female gender and sharing a household with children aged 0–9 years were associated with lower carriage. The most frequent genogroups were capsule null locus (*cnl*), group B and group Y and the most commonly identified clonal complexes (cc) were cc198 and cc23. Persistent carriage with the same meningococcal strain for 12 months was observed in two students. Follow-up times exceeding 12 months are recommended for future studies investigating long-term carriage of *N. meningitidis*.

## Introduction

The Gram-negative bacterium *Neisseria meningitidis* (also known as meningococcus) causes significant morbidity and mortality worldwide with disease classically presenting as rapidly progressing septicaemia and/or meningitis. Classification of *N*. *meningitidis* is based on the composition of its capsular polysaccharides. Twelve different capsular groups have been defined, with six of them (A, B, C, Y, W and X) predominating among cases of invasive disease [1]. Meningococcal isolates can be further characterised into clonal complexes (cc) using multilocus sequencing typing (MLST) [2], and genetically divided by whole-genome sequencing (WGS) [3]. Certain meningococcal lineages are more prone to cause disease rather than carriage, for example the hyperinvasive lineages belonging to cc11, cc32 and cc269 [3]. Carriage isolates may lack capsule which is a major virulence factor for *N*. *meningitidis* [4].

Humans are the only known reservoir of *N*. *meningitidis*. It is part of the normal oropharyngeal microbiota in healthy humans, and can be carried without symptoms or harm [5]. Invasive meningococcal disease (IMD) is rare compared to carriage, and is more common in newly colonised individuals [6]. IMD usually occurs sporadically, but the meningococcus is also associated with outbreaks [7]. The relationship between carriage and development of invasive disease is not fully understood, although several factors associated with carriage of *N. meningitidis* have been identified; including age, male sex, exposure to cigarette smoke, recent respiratory tract infections and closeness of social contacts [8–13].

Carriage rates of *N*. *meningitidis* vary with setting and age, and in recent years, a trend towards lower carriage rates among teenagers has been observed [14, 15]. The highest carriage rates are usually found among teenagers and young adults, with a peak incidence at 19 years of age according to a systematic review by Christensen *et al*. [16]. Several outbreaks of IMD have been reported from settings where teenagers and young adults gather, for example at youth camps, universities and military service [17, 18]. Increasing carriage prevalence have been reported among students the first weeks after admission to university [19, 20], indicating that socialising patterns are important for the dynamics of meningococcal carriage. Previous studies with repeated sampling have shown carriage of the same meningococcal strain for up to 8 months, but the upper limit of long-term carriage is still unknown [21, 22].

In Sweden since 2016, *N*. *meningitidis* group W (MenW) and group Y (MenY) have emerged as the most common causes of IMD in the country. It is unclear whether this change in group distribution is caused by certain bacterial virulence factors or is an effect of high carriage of these groups within the population.

The aims of this study were to examine *N. meningitidis* carriage among students at a university in Sweden with regard to carriage prevalence, risk factors for carriage, characteristics of carriage isolates and persistence of meningococcal carriage up to 12 months.

## Materials and methods

### Inclusion of participants

Study participants were recruited at Örebro University, Sweden, on four occasions, September 2018, January 2019, May 2019 and September 2019. The sample collection took place at two campuses of the university, the medical campus and the general campus, located at a distance of three km from each other.

All students at the university were eligible for inclusion and the students could participate on one or more of the sampling occasions. The students were informed about the study in advance via posters, social media and the university's home page. They were also approached about participation by the researchers in major hallways of the university buildings, and provided with written and oral information about the study. At inclusion, each participant generated a unique digital study code, enabling repeated data input from the same individual without storage of civic number or other personal data.

The participating students completed an electronic questionnaire, connected with their personal study code, to assess risk factors for meningococcal carriage, including age, household conditions, current or recent upper respiratory tract infection, recent use of antibiotics, smoking habits, frequency of alcohol drinking, frequency of attending parties, pubs or night clubs and prior meningococcal vaccination.

The questionnaire also contained questions about number of persons kissed in the last week, and whether the participant had shared a glass or bottle, as well as use of a water pipe or cannabis, which are both usually shared between people. In addition, the participants reported their use of Swedish snus (moist powder tobacco in powder form or packaged in tea bag-like portion sachets made of cellulose fibres, placed inside the upper lip).

### Ethical considerations

Prior to inclusion, the study participants digitally signed a written informed consent form which was provided electronically through the study website in such a way that it was not possible to generate the digital study code without giving informed consent. The study code was based on hash values unique to each individual but without any personal data, making participation in the study anonymous. This meant that the researchers did not have access to actively contact the students, leaving participation and re-participation fully in the hands of the volunteering students. The participants were offered coffee/tea with cookies and chocolates or sweets in acknowledgement of their participation. The study was approved by the Regional Ethical Review Board in Uppsala, Sweden (reference number 2017/499).

### Sample collection and microbiological methods

Throat swabs from the oropharyngeal area and from one tonsil were performed by experienced health care professionals using flocked plastic swabs (ESwab, Copan Diagnostics, Murrieta, CA, USA). Samples were placed in the ESwab tubes containing Amies transport media (Copan Diagnostics), and transported within 8 h to the national reference laboratory for *N. meningitidis* at Örebro University Hospital, where a duplex PCR targeting *ctrA* and *crgA* [23] was performed within 24 h. If found to be positive for both or either of *ctrA* or *crgA*, samples were subsequently cultured on selective and non-selective agar plates (GC agar with added vancomycin, colistin, nystatin and trimethoprim; and plain GC agar, respectively) incubated for 24 h in a humid CO_2_-enriched (5%) atmosphere at 36 ± 1 °C. If no visible growth was noticed after 24 h, the plates were incubated for an additional 24 h. *N. meningitidis* was confirmed by colony appearance, oxidase positivity and MALDI-TOF mass spectrometry (Bruker Daltonik GmbH, Bremen, Germany) and then subcultured and preserved.

The *ctrA*/*crgA* PCR used in this study was originally developed to detect invasive meningococcal isolates from fluids normally considered sterile. As a confirmatory analysis of the *ctrA*/*crgA* PCR, all collected samples were additionally analysed with a second PCR targeting *sodC* and *porA*, adapted from a previous carriage study [24]. Samples were considered as true positive if the culture showed growth of *N. meningitidis*, or showed positivity in both the *ctrA*/*crgA* and *sodC*/*porA* duplex PCRs (but not necessarily for all four PCR targets).

Except for the first sample occasion, an additional throat swab was performed that was placed in a DNA/RNA Shield Collection Tube (Zymo Research, Irvine, CA, USA) and stored for future RNA analyses.

### Molecular characterisation of meningococcal isolates

All culture-positive isolates were further characterised using WGS on the Illumina platform to define genogroup, MLST lineages and genetic similarities, as previously described [25]. However, from each culture-positive sample only one colony was characterised.

A phylogenetic tree of carriage isolates was created with BIGSdb integrated in the *Neisseria* PubMLST database [26] using the genome comparator tool (based on 1605 *N. meningitidis* core loci) and visualised with the online tool iTOL [27].

*N. meningitidis* detected by PCR alone, and where culture isolate was lacking, were genogrouped using multiplex real-time PCR for the groups A, B, C, W, Y and X according to the instructions provided by the manufacturer (Rotor-Gene Q, Venlo, The Netherlands).

### Statistical analyses

This was a descriptive study of *N. meningitidis* carriage, with cross-sectional data obtained at the four sampling occasions and with longitudinal data for students participating more than once. Logistic regression was used to adjust for possible confounders in the analysis of associations for risk factors and carriage. The effect of individuals participating more than once was balanced by using logistic regression with generalised estimating equations and with unstructured correlation structure between sampling occasions. Both unadjusted and adjusted analyses were performed to evaluate the variables' independent associations with carriage. All risk factors for carriage were analysed as categorical variables; however, age was analysed both as a continuous variable using odds ratio (OR) per year and in three categories (≤22, 23–29, ≥30 years). In addition, the frequencies of a number of habits (cigarette smoking, use of Swedish snus, alcohol drinking and attending parties, pubs or clubs) were analysed as linear trend (OR per category) in relation to carriage. The statistical calculations were performed using version 17 of STATA (StataCorp LLC, College Station, TX, USA). Two-sided *P* values of <0.05 were considered to be statistically significant.

## Results

### Participants' characteristics

**Fig. 1.**
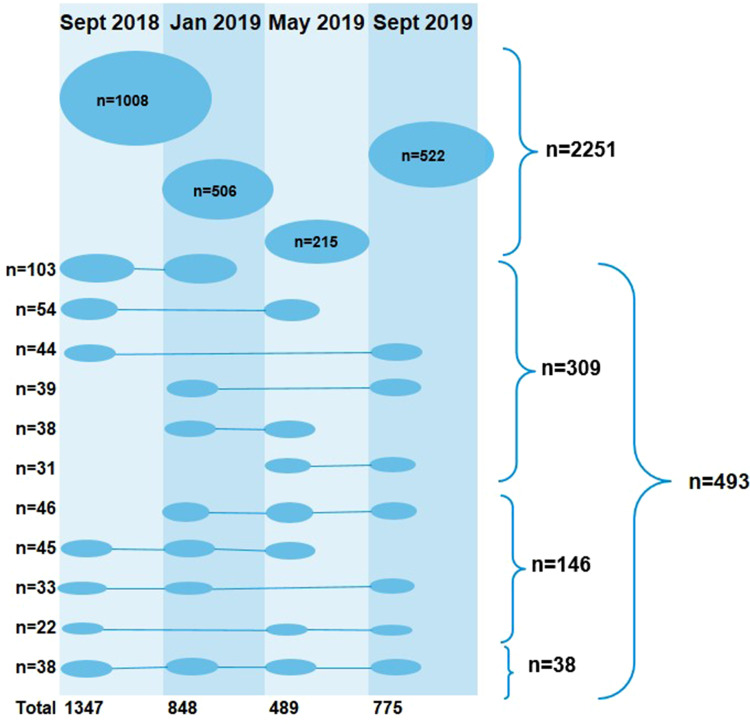
Distribution of students (*n* = 2744) participating in different combinations of the four sample occasions. Right-hand side: numbers of students participating once (*n* = 2251), twice (*n* = 309), three times (*n* = 146), and four times (*n* = 38).

A total of 2744 university students participated with both throat swabs and completed questionnaires, and hence were eligible for the risk factor analysis. Of these, 2251 (82.0%) participated once, 309 (11.3%) twice, 146 (5.3%) three times and 38 (1.4%) at all four occasions (Fig. 1).

In total, 3459 throat samples with accompanying questionnaires were collected. During the first sampling round in September 2018, 1347 students participated, followed by 848, 489 and 775, respectively, at subsequent occasions. A diverse pattern of participation was seen for the students who participated repeatedly (Fig. 1).

The characteristics of the participants are described in Table 1. The median age was 23 years (IQR: 21–27 years, range: 16–59 years) and 63.3% were female. Of the 3459 samples, 895 (25.9%) were obtained from students who lived alone, 2350 (67.9%) from students who lived with another adult, 422 (12.2%) from students who lived with children aged 0–9 years and 362 (10.5%) from students who lived with one or more children aged 10–19. A total of 203 students (5.8%) lived in a student corridor or other shared accommodation with more than two adults.

**Table 1.** Characteristics of participating students assessed for carriage of *N*. *meningitidis*, presented data that applied during sampling of the 3459 samples

		*n* (%)
Female gender		2185 (63.3)
Age in years	≤22	1410 (40.8)
	23–29	1495 (43.2)
	≥30	554 (16.0)
Household members (one or more)	Adult	2350 (67.9)
	Children 0–9 years	422 (12.2)
	Children 10–19 years	362 (10.5)
Previous tonsillectomy		257 (7.4)
Previous meningococcal vaccination		82 (2.4)
Travelled abroad^a^		677 (19.6)
Use of antibiotics^a^		207 (6.0)
Upper respiratory tract infection^b^		1166 (33.7)
Cigarette smoking	No	3210 (92.8)
	1–5 cigarettes/day	175 (5.1)
	≥6 cigarettes/day	74 (2.1)
Exposed to passive cigarette smoking		205 (5.9)
E-cigarette smoking^b^		40 (1.2)
Use of water pipe or cannabis^a^		276 (8.0)
Use of Swedish snus	No	2987 (86.4)
	1–5 portions/day	170 (4.9)
	≥6 portions/day	302 (8.7)
Alcohol drinking^b^	No	1298 (37.5)
	Once	1111 (32.1)
	Twice or more	1050 (30.4)
Shared a glass or bottle^b^		1479 (42.8)
Attended a party, pub or club^b^	No	1831 (52.9)
	Once	941 (27.2)
	Twice or more	687 (19.9)
Number of persons kissed^b^	None	1500 (43.3)
	1 person	1794 (51.9)
	≥2 persons	165 (4.8)

a In the past month.

b In the past week.

Smoking was recorded in 249 (7.2%) of the participating students and 472 (13.6%) used Swedish snus. A total of 82 (2.4%) participants reported having previously received any type of meningococcal vaccine.

### Carriage rate and risk factors for carriage

**Fig. 2.**
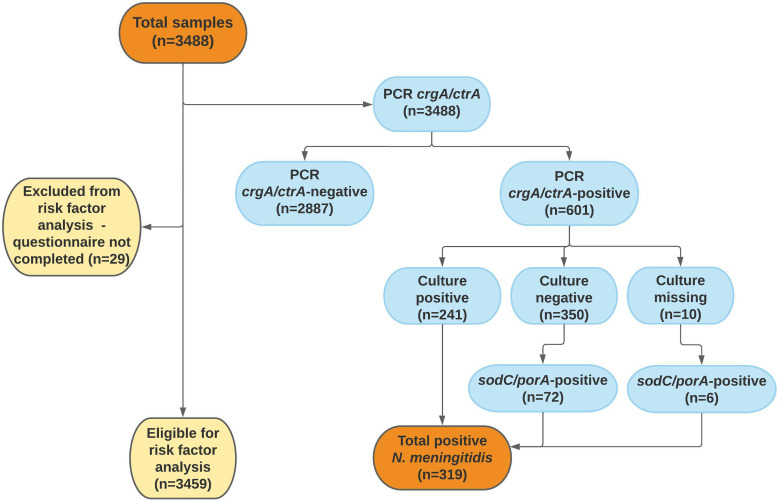
Flow chart of throat samples in the analysis of carriage of *N. meningitidis*. In 10 samples, culture was missed due to human error.

In addition to the 3459 samples with accompanying questionnaires, another 29 samples were collected but without a completed questionnaire. All 3488 samples were initially analysed using the *ctrA*/*crgA* PCR, of which 601 samples were positive and further cultured. In 241 samples, *N*. *meningitidis* was isolated. In another 78 samples, presence of *N*. *meningitidis* DNA was verified using the *sodC*/*porA* PCR, leading to a total carriage prevalence of 9.1% (95% CI 8.2–10.1, 319/3488; Figure 2). The group of students participating more than once (*n* = 493) provided 1208 samples, of which 8.4% (95% CI 7.0–10.1, 102/1208) were positive for *N*. *meningitidis*. Among students who participated only once (*n* = 2251), the carriage rate was 9.4% (95% CI 8.3–10.7, 212/2251). No statistical difference in carriage prevalence was seen between sampling occasions (Table 2). Culture was performed on a number of randomly selected *ctrA*/*crgA* negative samples (*n* = 291); all of these were culture-negative.

**Table 2.** Unadjusted and adjusted analysis of risk factors for carriage of *N. meningitidis* for samples with accompanying questionnaire (carriers of *N*. *meningitidis*: 314/3459, 9.1%; 95% CI 8.2–10.1)

			Unadjusted (*n* = 3459)		Adjusted (*n* = 3453)	
Variables	Categories	Number of carriers (%)	OR (95% CI)	*P*	OR (95% CI)	*P*
Sampling session	September 2018	128 (9.5)	Ref		Ref	
	January 2019	77 (9.1)	0.87 (0.66–1.14)	0.31	0.96 (0.72–1.28)	0.77
	May 2019	40 (8.2)	0.87 (0.65–1.17)	0.35	0.91 (0.65–1.26)	0.55
	September 2019	69 (8.9)	0.87 (0.66–1.14)	0.33	0.94 (0.70–1.25)	0.66
Gender^a^	Male	165 (13.0)	Ref		Ref	
	Female	149 (6.8)	***0.54*** (***0.42–0.70)***	***<0***.***01***	***0.60*** (***0.46–0.80)***	***<0***.***01***
Age	Per year		***0.92*** (***0.90–0.95)***	***<0***.***01***		
	≤22 years	158 (11.2)	Ref		Ref	
	23–29 years	131 (8.8)	***0.77*** (***0.60–0.99)***	***0***.***04***	***0.75*** (***0.57–0.98)***	***0***.***03***
	≥30 years	25 (4.5)	***0.41*** (***0.27–0.63)***	***<0***.***01***	0.71 (0.43–1.17)	0.18
Another adult in the household	No	118 (10.6)	Ref		Ref	
	Yes	196 (8.3)	***0.76*** (***0.59–0.98)***	***0***.***03***	0.89 (0.68–1.16)	0.38
Children 0–9 years in the household	No	305 (10.0)	Ref		Ref	
	Yes	9 (2.1)	***0.22*** (***0.11–0.44)***	***<0***.***01***	***0.32*** (***0.16–0.67)***	***<0***.***01***
Children 10–19 years in the household	No	291 (9.4)	Ref		Ref	
	Yes	23 (6.3)	0.68 (0.46–1.02)	0.06	0.89 (0.57–1.38)	0.60
Previous tonsillectomy	No	281 (8.8)	Ref		Ref	
	Yes	33 (12.8)	***1.65*** (***1.10–2.47)***	***0***.***01***	***1.64*** (***1.09–2.49)***	***0***.***02***
Previous meningococcal vaccination	No	311 (9.2)	Ref		Ref	
	Yes	3 (3.7)	0.59 (0.21–1.63)	0.31	0.54 (0.18–1.62)	0.27
Travelled abroad^b^	No	243 (8.7)	Ref		Ref	
	Yes	71 (10.5)	***1.29*** (***1.01–1.64)***	***0***.***04***	1.22 (0.93–1.60)	0.15
Use of antibiotics^b^	No	296 (9.1)	Ref		Ref	
	Yes	18 (8.7)	0.89 (0.56–1.40)	0.61	0.81 (0.51–1.31)	0.39
Upper respiratory tract infection^c^	No	193 (8.4)	Ref		Ref	
	Yes	121 (10.4)	1.10 (0.86–1.41)	0.44	0.95 (0.73–1.23)	0.70
Cigarette smoking	No	281 (8.8)	Ref		Ref	
	1–5 cigarettes/day	22 (12.6)	1.22 (0.63–2.36)	0.56	1.16 (0.67–2.01)	0.60
	≥6 cigarettes/day	11 (14.9)	***1.91*** (***1.01–3.64)***	***0***.***04***	***2.36*** (***1.18–4.75)***	***0***.***02***
	Linear trend, OR per category		1.33 (0.96–1.84)	0.09	***1.39*** (***1.00–1.93)***	***0***.***04***
	Yes	33 (13.2)	1.40 (0.83–2.33)	0.20		
Exposed to passive cigarette smoking	No	297 (9.1)	Ref		Ref	
	Yes	17 (8.3)	0.83 (0.49–1.42)	0.50	0.69 (0.40–1.19)	0.18
E-cigarette smoking^c^	No	307 (9.0)	Ref		Ref	
	Yes	7 (17.5)	1.46 (0.38–5.56)	0.58	0.96 (0.31–2.94)	0.94
Use of water pipe or cannabis^b^	No	282 (8.9)	Ref		Ref	
	Yes	32 (11.6)	1.35 (0.95–1.93)	0.10	1.07 (0.73–1.57)	0.74
Use of Swedish snus	No	244 (8.2)	Ref		Ref	
	1–5 portions/day	28 (16.5)	***1.69*** (***1.02–2.80)***	***0***.***04***	1.43 (0.89–2.31)	0.14
	≥6 portions/day	42 (13.9)	***1.75*** (***1.22–2.50)***	***<0***.***01***	1.35 (0.90–2.01)	0.14
	Linear trend, OR per category		***1.36*** (***1.14–1.61)***	***<0***.***01***	1.19 (0.98–1.44)	0.08
	Yes	70 (14.8)	***1.72*** (***1.26–2.36)***	***<0***.***01***		
Drinking alcohol^c^	No	64 (4.9)	Ref		Ref	
	Once	107 (9.6)	***1.67*** (***1.24–2.24)***	***<0***.***01***	***1.39*** (***1.00–1.92)***	***0***.***04***
	Twice or more	143 (13.6)	***2.35*** (***1.69–3.26)***	***<0***.***01***	1.35 (0.91–2.01)	0.14
	Linear trend, OR per category		***1.51*** (***1.28–1.77)***	***<0***.***01***	1.14 (0.94–1.39)	0.17
	Yes	250 (11.6)	***2.03*** (***1.54–2.67)***	***<0***.***01***		
Shared a glass or bottle^c^	No	145 (7.3)	Ref		Ref	
	Yes	169 (11.4)	***1.36*** (***1.08–1.73)***	***<0***.***01***	0.93 (0.72–1.18)	0.54
Attended a party, pub or club^c^	No	102 (5.6)	Ref		Ref	
	Once	95 (10.1)	***1.63*** (***1.15–2.33)***	***<0***.***01***	1.31 (0.96–1.81)	0.09
	Twice or more	117 (17.0)	***2.54*** (***1.83–3.52)***	***<0***.***01***	***2.00*** (***1.35–2.95)***	***<0***.***01***
	Linear trend, OR per category		***1.59*** (***1.35–1.88)***	***<0***.***01***	***1.41*** (***1.16–1.72)***	***<0***.***01***
	Yes	212 (13.0)	***2.03*** (***1.52–2.73)***	***<0***.***01***		
Number of persons kissed^c^	None	137 (9.1)	Ref		Ref	
	1 person	159 (8.9)	0.99 (0.78–1.27)	0.97	1.03 (0.80–1.33)	0.82
	≥2 persons	18 (10.9)	0.87 (0.43–1.74)	0.69	0.83 (0.45–1.54)	0.56

In the adjusted analysis, all variables were included in the model. Logistic regression as generalised estimating equations with panel samples, outcome meningococcal carriage, 2744 subjects. Significant results (*P* < 0.05) highlighted in bold and italic.

a Participants without specified gender (*n* = 6) not included in the adjusted analysis.

b In the past month.

c In the past week.

Among the 3459 samples with accompanying questionnaires that were eligible for risk factor analysis, the carriage rate was 9.1% (95% CI 8.1–10.0, 314/3459). In the unadjusted analysis, the following risk factors were significantly associated with higher carriage for *N*. *meningitidis*: previous tonsillectomy, travelling abroad in the past month, daily use of cigarettes or Swedish snus, drinking alcohol in the past week, sharing a glass/bottle in the past week and attending a party, pub or club in the past week. Female gender, age >22 years, living with another adult and living with children aged 0–9 years were associated with a lower risk of meningococcal carriage (Table 2).

In the adjusted analysis that included all variables, the following factors were significantly associated with higher carriage rates of *N*. *meningitidis*: previous tonsillectomy, daily cigarette smoking, drinking alcohol in the past week and attending a party, pub or club in the past week. Female gender, age >22 years and living with children aged 0–9 years were associated with lower risk. The analysis of age as a continuous variable revealed an odds reduction for meningococcal carriage of 8% per year of increasing age. A dose dependency was found between the daily number of cigarettes smoked and the risk of meningococcal carriage (OR per category: 1.39, 95% CI 1.00–1.93). In addition, a dose dependency was noted for the frequency of attendance at a party, pub or club during the week before sampling (OR per category: 1.41, 95% CI 1.16–1.72). The use of Swedish snus was not significantly associated with meningococcal carriage in the adjusted analysis (Table 2).

### Characterisation of carriage isolates

**Fig. 3.**
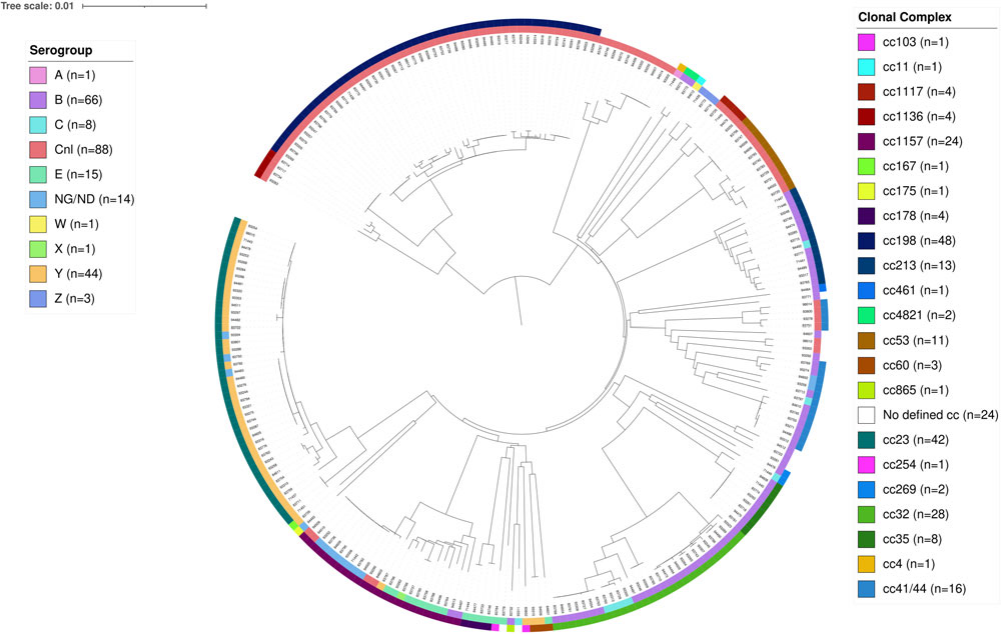
Phylogenetic relationships of *N. meningitidis* carriage isolates (*n* = 241) presented as a circular phylogram with the corresponding clonal complexes in the outermost circle, and the corresponding groups in the second outermost circle. Cnl = capsule null locus, NG/ND = non-groupable / no data. The scale of the tree indicates 1% genes with allele differences out of the analysed 1422 genes.

Of the 319 samples containing *N. meningitidis*, 241 culture isolates were further analysed for genogroup belonging using WGS, and 78 samples identified only with PCR were analysed using the multiplexed genogrouping PCR. The groups identified among the 241 culture isolates were capsule null locus (*cnl*) (*n* = 88), group B (*n* = 66), group Y (*n* = 44), group E (*n* = 15), group C (*n* = 8), group Z (*n* = 3), group A (*n* = 1), group W (*n* = 1) and group X (*n* = 1), whereas 14 isolates were non-groupable. The groups identified among the 78 only PCR positive samples were group B (*n* = 15), group Y (*n* = 10), group C (*n* = 2). In 51 of the samples analysed with the multiplex genogrouping PCR, none of the groups A, B, C, W, Y or X could be detected, and further characterisation of these samples in terms of, for example, *cnl* or non-groupable (NG) affiliation was not possible due to the low amount of DNA in some samples. The most common cc of the 241 samples analysed with WGS were ST-198 (*n* = 48), ST-23 (*n* = 42, of which 24 belonged to sequence type 23), ST-32 (*n* = 28, of which 13 belonged to sequence type 7460) and ST-1156 (*n* = 24) (Fig. 3). There were no significant differences in the distribution of genogroups or cc between the different sampling occasions.

### Long-term carriage

Table 3 presents data on the 32 students who were positive for *N. meningitidis* on more than one sampling occasion. In 20 of these students, the same meningococcal strain was found, while different strains over time were found in 10 students. In 6 students who were carriers more than once, the genogrouping analysis could not distinguish between shift or persistence of the meningococcal strain. Over the whole study period of 12 months, two individuals were persistent carriers of an identical strain verified by WGS (student numbers 1 and 11 in Table 3). One additional individual (student number 2 in Table 3) was a carrier of MenY for 12 months, but it was not possible to analyse one of the samples with WGS for exact strain determination.

**Table 3.** Study participants with more than one sample positive for carriage of *N*. *meningitidis*

Student number	September 2018	January 2019	May 2019	September 2019
1	B, ST-7460, cc32	n.a.	n.a.	B, ST-7460, cc32
2	Y, ST-23, cc23	Y, ST-23, cc23	Y, ST-23, cc23	Y
3	cnl, ST-823, cc198	cnl, ST-823, cc198	n.a.	n.a.
4	non-ABCWYX	non-ABCWYX	n.a.	n.a.
5	B, ST-3327, cc865	non-ABCWYX	n.a.	n.a.
6	cnl, ST-823, cc198	cnl, ST-823, cc198	n.a.	n.a.
7	B, ST-7460, cc32	n.a.	B, ST-7460, cc32	n.a.
8	Y	B	Negative	n.a.
9	cnl, ST-53, cc53	Y	Negative	n.a.
10	Z, ST-3882	B	n.a.	n.a.
11	B, ST-809, cc35	B, ST-809, cc35	B, ST-B, ST-809, cc35	B, ST-809, cc35
12	Negative	B	Negative	non-ABCWYX
13	B, ST-213, cc213	B, ST-213, cc213	Negative	n.a.
14	B	B	non-ABCWYX	n.a.
15	NG, ST-1649, cc1157	NG, ST-1649, cc1157	n.a.	n.a.
16	B, ST-7460, cc32	Negative	cnl, ST-7129	n.a.
17	NG, ST-1649, cc1157	NG, ST-1649, cc1157	n.a.	n.a.
18	non-ABCWYX	n.a.	non-ABCWYX	n.a.
19	B, ST-32, cc32	B, ST-32, cc32	n.a.	n.a.
20	Y, ST-11293, cc23	Y	n.a.	non-ABCWYX
21	n.a.	Y, ST-23, cc23	Y, ST-23, cc23	Y, ST-23, cc23
22	n.a.	E, ST-178, cc178	B	E, ST-178, cc178
23	n.a.	Negative	Y, ST-23, cc23	Y, ST-23, cc23
24	n.a.	cnl, ST-15911	cnl, ST-15911	cnl, ST-15911
25	n.a.	Y, ST-1655, cc23	non-ABCWYX	Y, ST-1655, cc23
26	n.a.	cnl, ST-823, cc198	n.a.	non-ABCWYX
27	n.a.	B, ST-7460, cc32	B, ST-7460, cc32	n.a.
28	n.a.	Y, ST-23, cc23	n.a.	Y
29	n.a.	B, ST-44, cc41/44	B, ST-44, cc41/44	n.a.
30	n.a.	n.a.	Y, ST-11293, cc23	Y, ST-11293, cc23
31	n.a.	n.a.	Y, ST-23, cc23	Y, ST-23, cc23
32	n.a.	n.a.	Y, ST-23, cc23	Y, ST-23, cc23

ST, sequence type; cc, clonal complex

n.a. = the student did not participate in the study period respectively.

Light green colour: persistence of same lineage confirmed by WGS.

Dark green colour: probably persistence of same lineage, the genogrouping methods used could not confirm if it was the same lineage at the different sampling times.

Blue colour: replacement of lineage or probably replacement of lineage.

## Discussion

The overall carriage rate for *N. meningitidis* was 9.1% among university students in Sweden with a median age of 23 years. Higher rates of meningococcal carriage were associated with previous tonsillectomy, daily cigarette smoking, drinking alcohol in the past week and attending parties, clubs and pubs in the past week, while lower carriage rate were associated with age ≥22 years, female gender and sharing a household with children aged 0–9 years. The most common cc were ST-198 and ST-23.

A number of previously known risk factors for meningococcal carriage were identified in this study, including smoking [28–30], with which the present study also confirmed a dose-dependent association. Both alcohol drinking and attending parties, pubs or clubs were associated with higher carriage rates, as seen in other studies [9, 30]. However, alcohol drinking and party attendance often occur simultaneously, making the causal relationship with carriage hard to assess. Previous tonsillectomy has been associated with higher carriage rates of *N. meningitidis* in other studies [31, 32], and this association was also seen in the present study. We studied one possible risk factor for meningococcal carriage that has been only sparsely studied before: the use of Swedish snus. A Norwegian study described an association between use of Swedish snus and meningococcal carriage [15], but we found no such association for the whole cohort in the adjusted analysis.

In this study, the participants were relatively old compared to carriage studies of university students in other countries. The older median age of the students might reflect social behaviours and hence, risk factors for meningococcal carriage.

This is the first meningococcal carriage study of this size to be performed in Sweden. In 1979, Kamme and Kahlmeter reported a carriage rate of 8% (27/332) among students at a Swedish university [33], but did not present any further characterisation of the isolates, and so it is not possible to evaluate how carriage of certain lineages or strains has developed over time in Sweden. Meningococcal carriage rates have decreased over recent decades in Norway [15] and in the UK [10]. One hypothesised explanation for this decrease is that change over time in social behaviours among teenagers and young adults has led to a trend of fewer social contacts, and hence fewer transmission opportunities for *N. meningitidis* [10].

In Sweden during the years when this study was conducted (2018–2019), the incidence of IMD was 0.6 cases per 100 000 inhabitants per year, and the most commonly identified groups among invasive isolates were MenW (36%, of which 37/44 belonged to the cc11 2013 strain), MenY (29%, of which 34/35 belonged to cc23) and MenB (13%) [34]. In this study, only one carriage isolate was grouped as MenW, belonging to the MenW cc11 2013 strain. The high incidence of MenW cc11 2013 strain invasive infections in Sweden may be due to high bacterial virulence, as studies on transgenic mice have shown that isolates of this particular strain caused higher levels of bacteraemia and induced more proinflammatory activity compared to MenY cc23 isolates [35]. Meningococcal carriage may induce bactericidal antibodies that are mainly strain-specific and would therefore not induce a general humoral defence against diverse meningococcal strains [36]. The low levels of MenW cc11 2013 strain carriage in this study may be associated with reduced strain-specific humoral immunity against this strain in the Swedish population, meaning that exposure to the MenW cc11 2013 strain is more likely to result in IMD rather than asymptomatic carriage. Similar findings have been reported from Chile, where Rubilar *et al*. noted a low carriage rate of group W ST-11 among adolescents in a year when the same clonal complex accounted for 66% of the total IMD cases in Chile that year [37]. In contrast, MenY cc23 was the second most common clonal complex among the carriage isolates in this study, and one of the most frequent causes of IMD during the study period. If representative of the Swedish population as a whole, the high carriage of MenY cc23 could be one possible explanation for the high frequency of MenY cc23 among invasive isolates during the same period in Sweden.

To our knowledge, this is the first report of carriage of the same meningococcal strain for 12 months or more as verified by WGS, which was found in two participants. Previous studies have shown meningococcal carriage of the same strain verified by WGS for up to 8 months [21, 22]. Other studies reported repeated meningococcal carriage for 14 months [38] and 18 months [39], respectively, but these reports lacked information on whether the same strain was preserved or whether there had been a strain replacement. Studies of meningococcal carriage over a longer period, using WGS or equivalent methods, are needed to assess how long carriage of the same meningococcal strain can last. Also, the role of persistent carriage should be studied further. Prolonged carriage of hypervirulent strains may put more people at risk for severe infections; however, long-time carriage of non-encapsulated meningococci may act protective for invasive disease for the individual.

One strength of this study was that the questionnaires were filled in electronically, yielding a high completion rate, though all data in the questionnaire were self-reported and not checked objectively. Another strength was the use of an individual study number that enabled repeated testing without saving any personal information, which facilitated privacy for the study participants. However, this approach prevented active follow up for repeated testing, which might have limited the study of long-term carriage as only a low number of students participated all four times. The choice of methods comprised a strength, with PCR for the identification of *N*. *meningitidis* in all samples yielding high sensitivity and specificity [24, 40–42], followed by culture of PCR-positive samples for further characterisation of isolates using WGS. One possible limitation was that only one colony from each culture-positive sample was further characterised with WGS or other molecular methods, and so we did not study the carriage of multiple meningococcal lineages or strains within the same individual. Another limitation was that the samples where *N*. *meningitidis* was detected by PCR only, and not cultured, were only further characterised using genogrouping PCR. For these samples, it was therefore not possible to determine if the study participant carried the same meningococcal strain at the different sampling times.

This study of meningococcal carriage was performed prior to the Covid-19 pandemic, and hence before any restrictions were imposed on social interactions. During the pandemic, the reported IMD cases dropped in many countries including Sweden [43]. It would be of great interest to study whether meningococcal carriage has changed during the pandemic and after communities have returned to previous social behaviours, regarding both the carriage rate per se and the distribution of pathogenic and non-pathogenic lineages.

## Conclusion

In this study, carriage of *N*. *meningitidis* was 9.1% and previously known risk factors for meningococcal carriage could be confirmed. About half of the identified *N. meningitidis* belonged to cc that are not associated with invasive disease.

With repeated testing and by using WGS, we found carriage of the same meningococcal strain over a period of 1 year, indicating long-term carriage. Further studies with longer follow-up times and smaller swabbing intervals are needed to better understand the dynamics of meningococcal carriage.

## Data Availability

The data underlying this study is available from the corresponding author upon reasonable request.
