# Prevalence and persistence of Neisseria meningitidis carriage in Swedish university students – CORRIGENDUM

**DOI:** 10.1017/S0950268824000840

**Published:** 2024-08-22

**Authors:** Olof Säll, Lorraine Eriksson, Idosa Asfaw Berhane, Alexander Persson, Anders Magnuson, Sara Thulin Hedberg, Martin Sundqvist, Per Olcén, Hans Fredlund, Bianca Stenmark, Eva Särndahl, Paula Mölling, Susanne Jacobsson

When this article was originally published, the author list was incorrect.

This has now been updated.
